# Bicondylar Hoffa fracture with concurrent medial and lateral collateral ligament avulsion: A case report

**DOI:** 10.1002/ccr3.7466

**Published:** 2023-06-02

**Authors:** Sajad Noorigaravand, Razieh Heidari, Hamed Tayyebi, Saeid Shirvani, Shayan Amiri

**Affiliations:** ^1^ Assistant professor of orthopedic surgery Shohadaye Haftom‐e‐Tir Hospital, school of medicine, Iran University of Medical Sciences Tehran Iran; ^2^ Department of radiology Iran university of medical sciences Tehran Iran

**Keywords:** approach, bicondylar Hoffa fracture, lateral collateral ligament, medial collateral ligament, surgery

## Abstract

Hoffa fractures are rare fractures of the femoral condyle that occur in the coronal plane of the bone. In most cases, high‐energy trauma leads to isolated coronal fractures of one of the femoral condyles, medial or lateral. Even with a typical unicondylar Hoffa fracture, our patient sustained a bicondylar Hoffa fracture in his right knee after falling from high and suffering direct trauma as well. The fracture was approached from both the medial and lateral sides of the distal femur. Three‐month follow‐up showed excellent functional scores, no laxity, and no pain.

## 1 INTRODUCTION

The isolated coronal plane fracture of the distal femoral condyle is a rare fracture described by Friedrich Busch (1844–1916)^1^ in 1869 and named after Albert Hoffa (1859–1907) in 1904.^2^ Only 0.65% of all femoral fractures occur in this way. It is often easy to diagnose, but on anteroposterior and lateral radiographs, Hoffa's fracture may be overlooked due to overlapping condyles.^3^ In spite of the preponderance of lateral condyle involvement, medial condyle involvement is not as common as previously thought.^4^ An axial transmission of ground reaction force directed posteriorly in a flexed knee joint is the typical mechanism of injury that results in lateral condylar split fractures. A bicondylar Hoffa is a very rare condition which may occur if these forces are transmitted to both condyles due to direct impact over the knee. As this fracture occurs within the articulation, conservative management is often accompanied by a risk of malunion, nonunion, instability, and post‐traumatic arthritis, which may compromise knee function.^5^, ^6^, ^7^ Therefore, current recommendations emphasize anatomical reduction and rigid internal fixation with lag screws or plates, according to fracture geometry and surgeon expertise.^7^, ^8^, ^9^ Those who suffer from “Type 4” fractures require individualized treatment based on their specific characteristics. It is denoted by “4b” because bicondylar involvement is involved. The fracture geometry, fragment size, and combination of these fractures can lead to two different treatment strategies. An anterior to posterior screw can suffice if the fragment is larger than 2.5 cm, while a posterior to anterior screw is required if the fragment is smaller. It has also been suggested that double incisions or the Swashbuckler approach be used.^10^ Anatomical reduction and internal fixation are now the accepted methods of treatment for these fractures, as opposed to nonoperative treatment in the past.^11^ In this case report, we describe a bicondylar Hoffa fracture with avulsion of both the LCL and MCL.

## 2 CASE PRESENTATION

**FIGURE 1:**
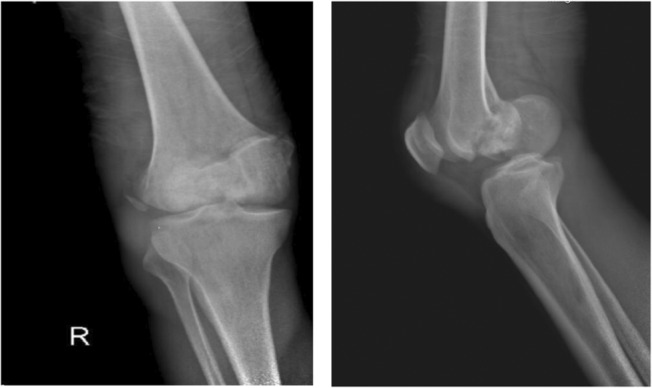
X‐rays of the right knee joint, anterior–posterior and medio‐lateral, showing a bicondylar Hoffa fracture.

**FIGURE 2:**
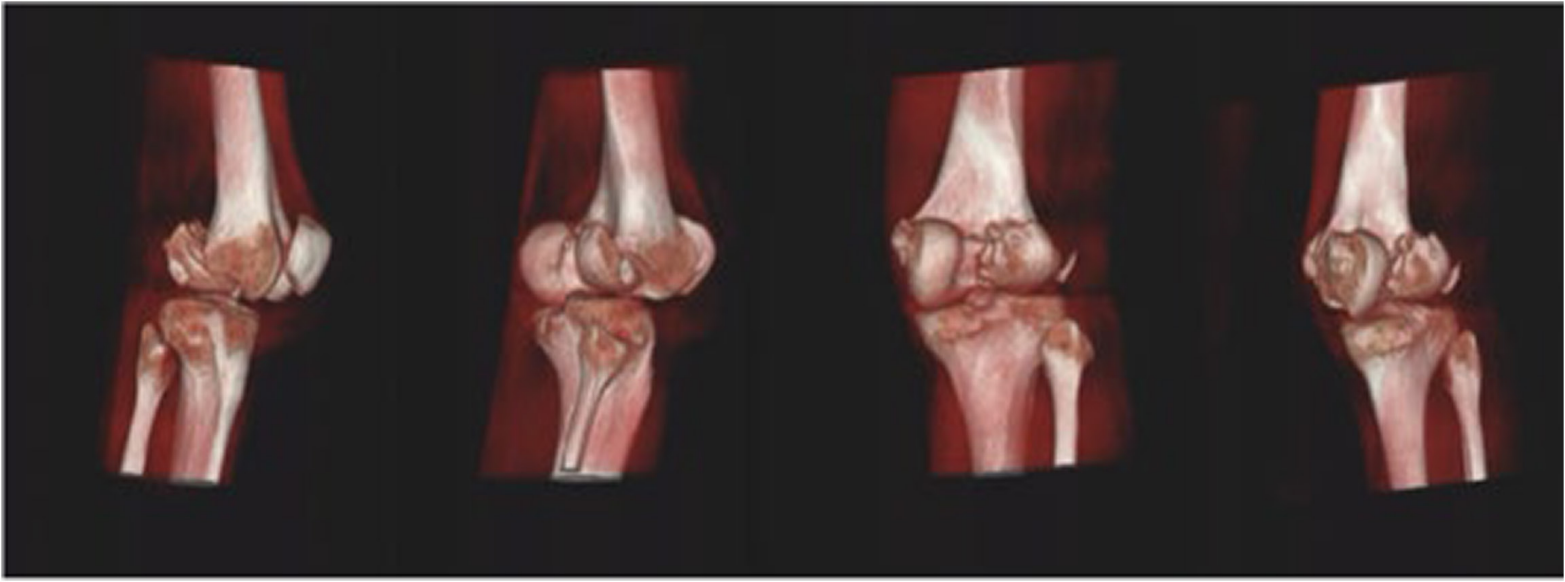
Medial and lateral femoral condyles fractures in the 3D‐CT‐scan.

**FIGURE 3:**
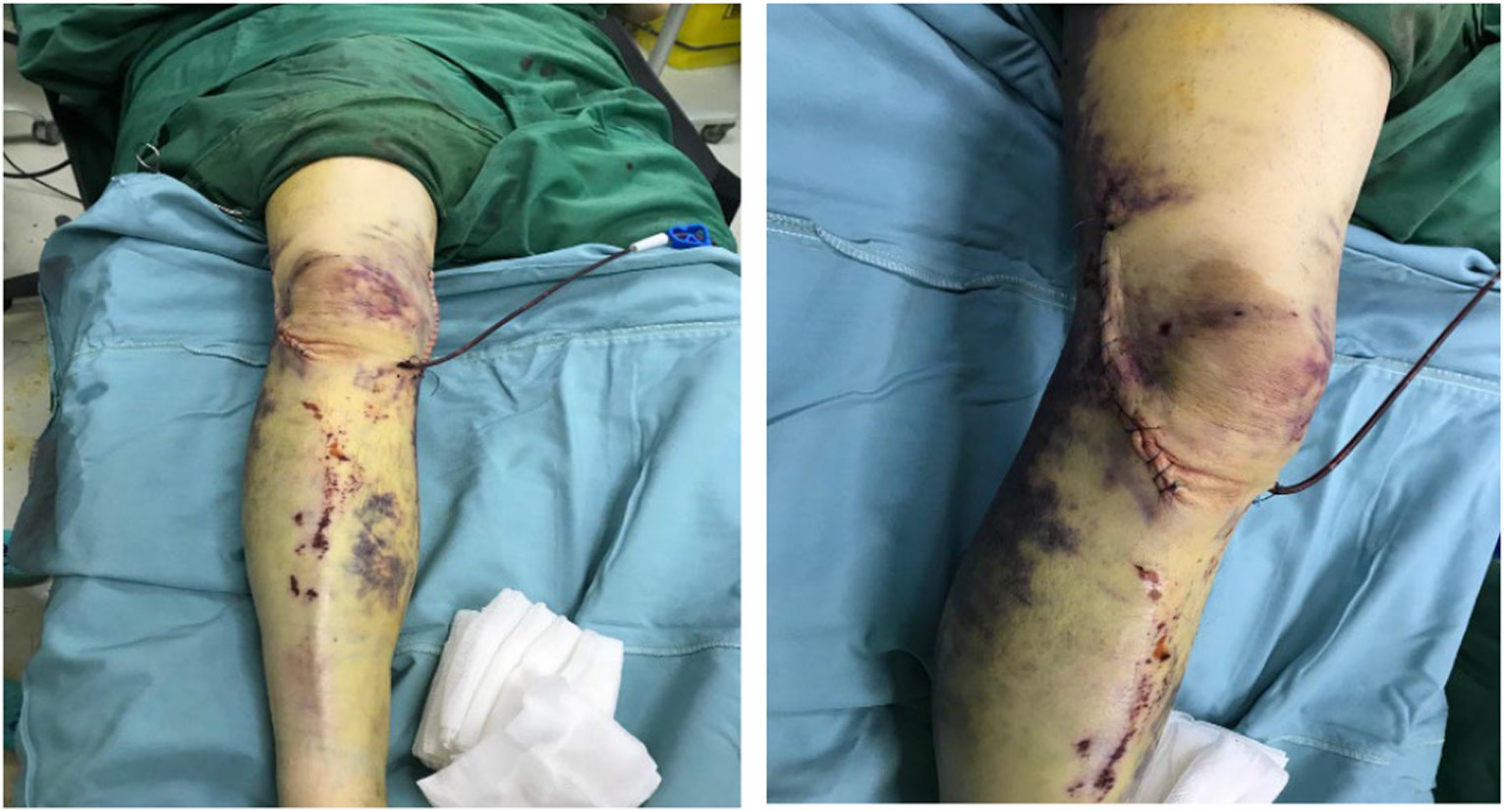
Operation's incisions.

**FIGURE 4:**
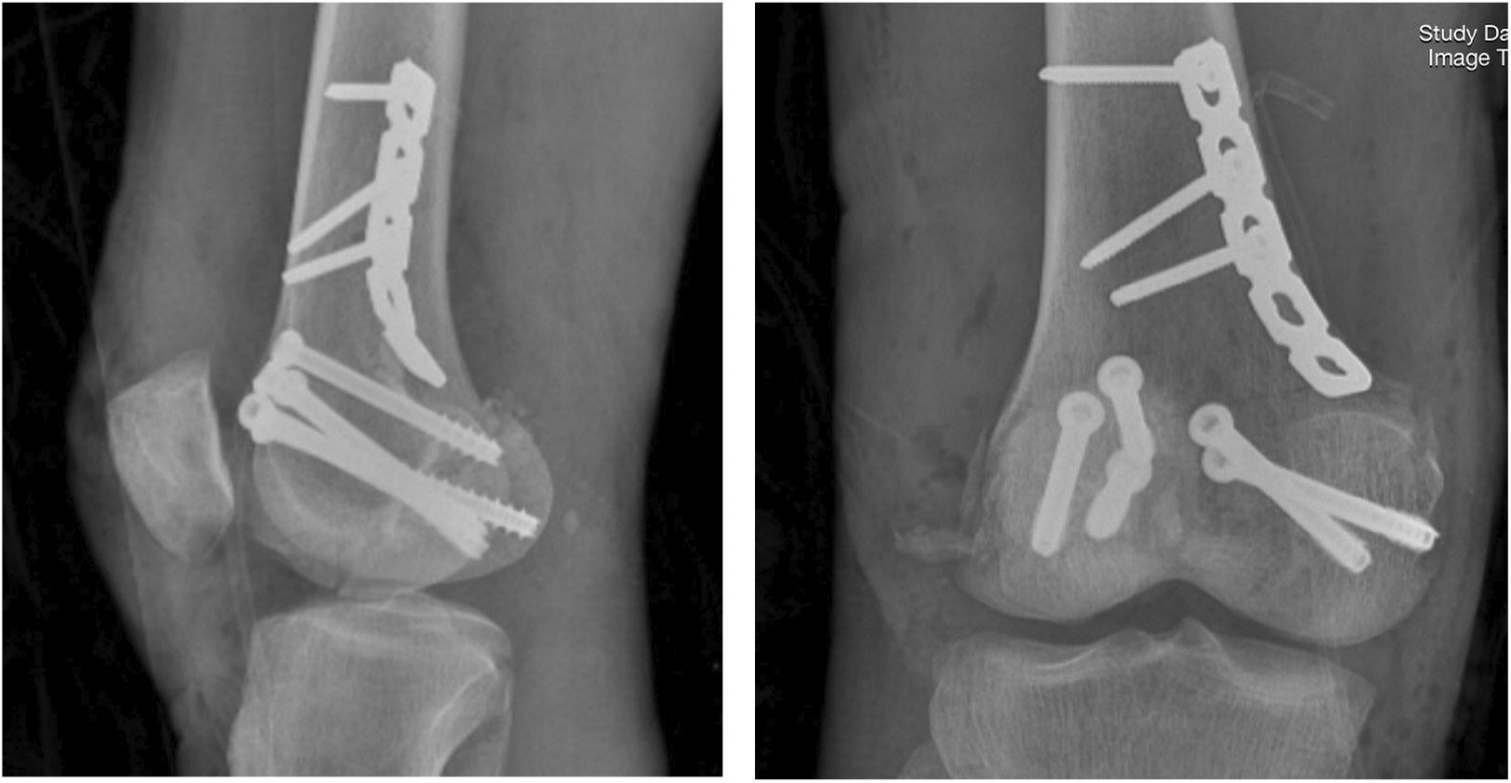
Postoperative radiograph of the right knee in anteroposterior and medio‐lateral views with cannulated cancellous screws.

**FIGURE 5:**
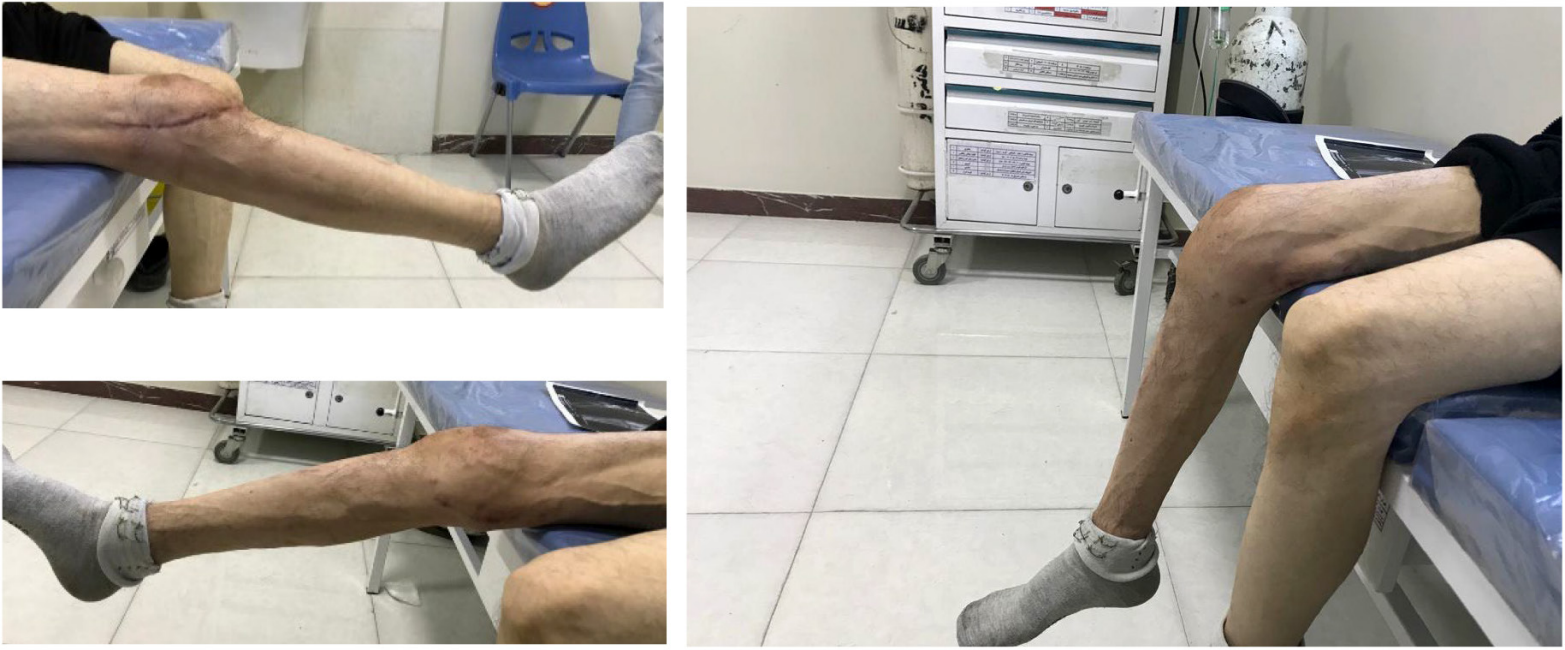
3 months after surgery, the patient's range of motion.

A 46‐year‐old man was admitted to the emergency department after falling from a building, which had been under construction, and after his fall he was injured by a falling brick in his right knee. A bicondylar Hoffa fracture was observed on the right knee based on radiographic examination (Figure 1) and computed tomography (Figure 2). Both the lateral and medial were displaced (Figure 3). The patient's radiographs and CT scans also revealed avulsions of the medial and lateral collateral ligaments. Upon examination after anesthesia, both varus and valgus were unstable. Therefore, we decided to do a double approach since we could fix collaterals as well as fractures. An open reduction and internal fixation were performed 2 days after the accident. The patient was positioned supine with exsanguinated right limb. We used both medial and lateral approaches to the distal femur by incision on both sides. A lateral parapatellar arthrotomy was performed in order to discharge the joint hematoma. A flexed knee was used to deliver the Hoffa fragments manually from the anterior. As a result of anatomical reduction and the placement of multiple Kirschner wires of 2 mm to stabilize and reduce the temporal defects (Figure 3), the fragments were then anatomically reduced. After that, a large, pointed reduction clamp was used to compress the fragments. A direct visual evaluation of the articular surface and fluoroscopy confirmed anatomical reduction. In the deep flexion position at the posterior articular surface, six (three per fragment) 6/5 cannulated screw were inserted over 1.4 mm Kirschner wires and directed anteriorly perpendicular to the fracture plane to compress the fractures (Figure 4). Just below the cartilage‐bone interface, these screws were driven in. In the final step, 3.5 mm reconstruction plate as an anti‐glide were applied in order to fix the fractures of the medial wall of the medial condyle and we used transosseous nonabsorbable suture to fix the medial and lateral collateral ligaments. We took post‐operation radiograph after the surgery (Figure 4). We have visited the patient in 2, 4, 8, 12 weeks after the surgery. In the first month post operation, intermittent knee mobilization and isometric muscle strengthening exercises were prescribed and he was also allowed to have toe‐touch weight bearing. The sutures were removed 2 weeks later post‐operation. In the fourth week after the surgery, the patient switched from a walker to a cane, and after 8 weeks, the patient was allowed to have full weight‐bearing. According to the latest follow‐up, the patient was able to do all her daily and work activities without discomfort at 3 months after surgery and the knee range of motion had a range of 0–100 (Figure 5).

## 3 DISCUSSION

In certain cases, a high‐energy trauma can result in a bicondylar Hoffa fracture, a condition that is very rare.^7^ Various factors contribute to the complexity of the anatomy of Hoffa fractures, including diagnostic difficulties, surgical exposure challenges, the inability to use conventional implants, as well as the weak and variable fixation constructs in this system, based on CT scan images that were used in the formation of this system, fracture patterns were classified as a result of their fault distribution according to the fracture pattern itself. Generally, a Type 1 fracture occurs at a point near the junction between the posterior condyle and the shaft of the femur, approximately coronally to the site of fracture, when the fracture line is located near this junction. Approximately 2.5 cm of the fragment extend from the posterior‐most point of the posterior condyle to the most anterior point of the fragment. It is known as Type 2 fracture if the fracture line passes anteriorly to the junction between the posterior femoral condyle and shaft, resulting in a 2.5 cm fragment. Usually, comminuted coronal fractures of the femur are seen in Type 3. A Type 4 fracture may be classified as one of four types: an anterior lip fracture Type 4a; a bicondylar fracture Type 4b; a marginal osteochondral fracture Type 4c; and a supracondylar fracture of the distal femur fracture Type 4d.^12^ Radiographs of anteroposterior and lateral Hoffa fractures can often be difficult to detect when they are not displaced. A CT scan may be necessary if fracture morphology is uncertain, according to an oblique radiograph.^3^ In order to assess the right knee, we obtain plain radiography (in frontal and lateral views) as well as a 3D‐CT scan (Figures 1 and 2). Figure 1 shows a Hoffa fracture that cannot be seen clearly, but Figure 2 shows it very clearly and as can be seen, it is bicondylar. Additionally, “Type 4” fractures are intrinsically unstable and require surgical fixation as a result of continuous shear stresses both coronal and sagittal. This type of fracture requires individual management depending on the severity of the injury. There was a Hoffa fracture in our case that was affecting the bicondylar region (4b). Depending on the fracture geometry, fragment size, and comminution, these fractures can be treated as two different entities. In cases where the fragment is larger than 2.5 cm, an anterior to posterior screw can suffice, while a posterior to anterior screw is essential in cases where the fragment is smaller. The Swashbuckler approach is also a viable option.^12^ Therefore, we use a 5 cm incision on both sides of the distal femur to perform a double incision. For this surgery, we used screws and wires in the deep flexion position, directed anteriorly perpendicular to the fracture plane in order to compress the fracture. After that, the fractures in the medial wall of the medial condyle were repaired with reconstruction plates as anti‐glide plates. In the absence of surgery, nonoperative treatments such as plaster casts or skeletal traction lead to a loss of extension, nonunion, instability, and deformity of the joints.^13^ Infectious hazards, vascular degeneration, and fragment necrosis have been reported with this surgical approach,^11^ but we avoided these concerns with antibiotic therapy and intensive care.

## 4 CONCLUSION

In conclusion, we describe a rare case of a bicondylar Hoffa fracture despite the fact that Hoffa fractures are usually unicondylar. Our team used both medial and lateral approaches to fix the fractures in the distal femur. A 3‐month follow‐up with the patient showed excellent results. He was able to walk with full weight bearing with comfort and had a satisfactory range of motion in his knee.

## AUTHOR CONTRIBUTIONS

**Sajad Noorigaravand:** Data curation; methodology; resources; supervision. **razieh heidari:** Investigation; methodology; resources; supervision. **Hamed Tayyebi:** Investigation; methodology; resources; supervision. **saied shirvani:** Data curation; investigation; methodology; resources; supervision. **Shayan Amiri:** Conceptualization; data curation; formal analysis; investigation; writing – original draft; writing – review and editing.

## FUNDING INFORMATION

None.

## CONFLICT OF INTEREST STATEMENT

The authors have no conflict of interest to declare.

## CONSENT

Written informed consent was obtained from the patient to publish this report in accordance with the journal's patient consent policy.

## Data Availability

Data will be made available on request.
